# Molecular characterization of haemosporidian and haemogregarine diversity in southwestern Iberian amphibians and reptiles

**DOI:** 10.1007/s00436-023-07814-6

**Published:** 2023-03-18

**Authors:** Daniel Parejo-Pulido, Carlos Mora-Rubio, Alfonso Marzal, Sergio Magallanes

**Affiliations:** 1 Instituto de Investigación en Recursos Cinegéticos (IREC), CSIC-UCLM-JCCM, Ronda de Toledo, 12, 13005 Ciudad Real, Spain; 2 Departamento de Anatomía Biología Celular y Zoología, Universidad de Extremadura, Avda. Elvas s/n, 06006 Badajoz, Spain; 3 Grupo de Investigaciones en Fauna Silvestre, Universidad Nacional de San Martín, Jr. Maynas 1777, 22021 Tarapoto, Perú; 4 Department of Wetland Ecology (EBD-CSIC), Estación Biológica de Doñana, Avda. Américo Vespucio 26, E-41092 Seville, Spain

**Keywords:** *Haemocystidium* sp., *Haemogregarina* sp., *Hepatozoon* sp., *Malpolon monspessulanus*, Phylogenetic relationship, *Podarcis virescens*, *Tarentola mauritanica*

## Abstract

The knowledge of the diversity and geographic distribution of parasite species is the first step towards understanding processes of global epidemiology and species conservation. Despite recent increases in research on haemosporidian and haemogregarine parasites of reptiles and amphibians, we still know little about their diversity and parasite-host interactions, especially in the Iberian Peninsula, where a few studies have been conducted. In this study, the haemosporidian and haemogregarine diversity and phylogenetic relationships of the parasites in southwestern Iberian amphibians and reptiles were assessed using PCR approaches on blood samples of 145 individuals from five amphibian and 13 reptile species. The amphibians did not present any of both groups of parasites studied. Regarding reptiles, five *Hepatozoon*, one *Haemogregarina*, and one *Haemocystidum* haplotypes were found infecting four different species, revealing new host records for these parasites. Among them, we found one new *Haemocystidium* haplotype and three new and a previously reported *Hepatozoon* haplotype from a north African snake. The latter finding suggests that some *Hepatozoon* parasites may not be host-specific and have large geographic ranges even crossing geographical barriers. These results increased the knowledge about the geographic distribution and the number of known host species of some reptile apicomplexan parasites, highlighting the great unexplored diversity of them in this region.

**Supplementary Information:**

The online version contains supplementary material available at 10.1007/s00436-023-07814-6.

## Introduction

Parasites are one of the most ubiquitous and abundant organisms in the world having a wide range of negative effects on their host life-histories (Schmid-Hempel 2011). Moreover, parasites play key roles not only on individual hosts, but also on many ecology interactions, host population dynamics, and community structure in many ecosystems (Price et al. 1986; Wood et al. 2007). In this scenario, the knowledge of the diversity and geographic distributions of parasite species at various spatial scales and host groups is the first step towards understanding processes of global epidemiology and species conservation (du Toit et al. 2013; Wells et al. 2015). The phylum Apicomplexa is a highly diverse protist group of obligatory parasitic organisms. The medical and veterinary importance of some of its genera (e.g., *Plasmodium*, *Toxoplasma*, and *Babesia*, among others) has contributed to the increase in the number of the studies on this group of parasites in the last two decades (Yabsley and Shock 2012; Santiago-Alarcón and Marzal 2020). Even so, the number of formally described species is far from the existing reality (Votýpka et al. 2016). In addition, many of these species were described on mammal and avian hosts, while studies of amphibians’ and reptiles’ apicomplexan diversity are even scarcer (Telford 2009).

Amphibians and reptiles are hosts to a wide variety of hemoparasitic apicomplexan groups, including hemococcidians, haemogregarines, haemosporidians, piroplasms, and Sarcocystidae (Davies and Johnston 2000; Telford 2009; Muriel et al. 2021). Haemogregarines of the genus *Hepatozoon* are the most common apicomplexans infecting these animals, although other haemogregarine genera such as *Haemogregarina*, *Hemolivia*, and *Karyolysus* can be also found (Smith 1996; Davies and Johnston 2000; Telford 2009). Haemogregarines have a heteroxenous life cycle with two different hosts: a vertebrate host in which asexual reproduction occurs, and an invertebrate vector in which sexual reproduction happens (Telford 2009). Recorded invertebrate vectors of apicomplexan parasites of reptiles and amphibians include ticks, mites, biting flies, mosquitoes, and leeches (Smith 1996). Likewise, haemosporidians of the genera *Plasmodium*, *Haemoproteus*, and *Haemocystidium* are also heteroxenous parasites with similar life cycles transmitted by hematophagous dipterans (Garnham 1966; Valkiūnas 2005). Among them, *Plasmodium* and *Haemocystidium* have been reported infecting reptiles, whereas *Haemoproteus* has been found in both amphibians and reptiles (Davies and Johnston 2000; Valkiūnas 2005; Telford 2009; Muriel et al. 2021). However, the genus *Haemocystidium* has not been fully accepted by the academic community due to its limited knowledge, and it was previously synonymized with the genus *Plasmodium* first and later with the genus *Haemoproteus* (Javanbakht et al. 2015; Austen et al. 2020). Nevertheless, molecular studies have shown that *Haemocystidium* species reported from reptiles are a distinct group from avian *Haemoproteus* and thus all reptilian *Haemoproteus* should be reclassified within the genus *Haemocystidium* (Maia et al. 2016a; Pineda-Catalan et al. 2013).

The establishment of molecular techniques for the detection of blood parasites (Vilcins et al. 2009) has revealed unexpectedly high levels of apicomplexan parasite diversity in amphibians and reptiles worldwide (O’Dwyer et al. 2013; Tomé et al. 2014; Harris et al. 2015). For example, many new lineages and species of *Hepatozoon* of certain regions and host species have been identified using these techniques (e.g., Maia et al. 2012; Rajabi et al. 2017; Gutiérrez-Liberato et al. 2021). In contrast, studies about amphibian and reptile haemosporidians are scarcer in the literature compared to those of haemogregarines (e.g., Fantham et al. 1942; Martinele et al. 2016; Matta et al. 2018). The pattern of distribution and genetic diversity of parasite species is shaped by the range of hosts in which they occur (Poulin et al. 2011). However, it is still unknown a large share of the diversity of amphibian and reptile haemogregarine and haemosporidian fauna from many regions and host species. Therefore, the screening of different species and in different countries is needed in order to reveal new lineages and new parasite-host interactions.

In the Iberian Peninsula, haemogregarine parasites have been reported from 14 species of reptiles such as *Algyrodes marchi*, *Psammodromus algirus*, or *Timon lepidus* (Amo et al. 2005a, 2007; Maia et al. 2012), and to a greater extent in species of the genus *Podarcis* (Amo et al. 2005b; Roca and Galdón 2010; Maia et al. 2012), while none from amphibians (Seabra-Babo et al. 2015). In contrast, to the best of our knowledge, no studies have been conducted on amphibian and reptile haemosporidians in this area. This study aimed to characterize the diversity of haemosporidian and haemogregarine parasites in some species of amphibians and reptiles in the southwestern Iberian Peninsula by using molecular approaches. Furthermore, the phylogenetic relationships of the haemosporidian and haemogregarine species detected with those found to date are analyzed.

## Materials and methods

### Sample collection

**Fig. 1:**
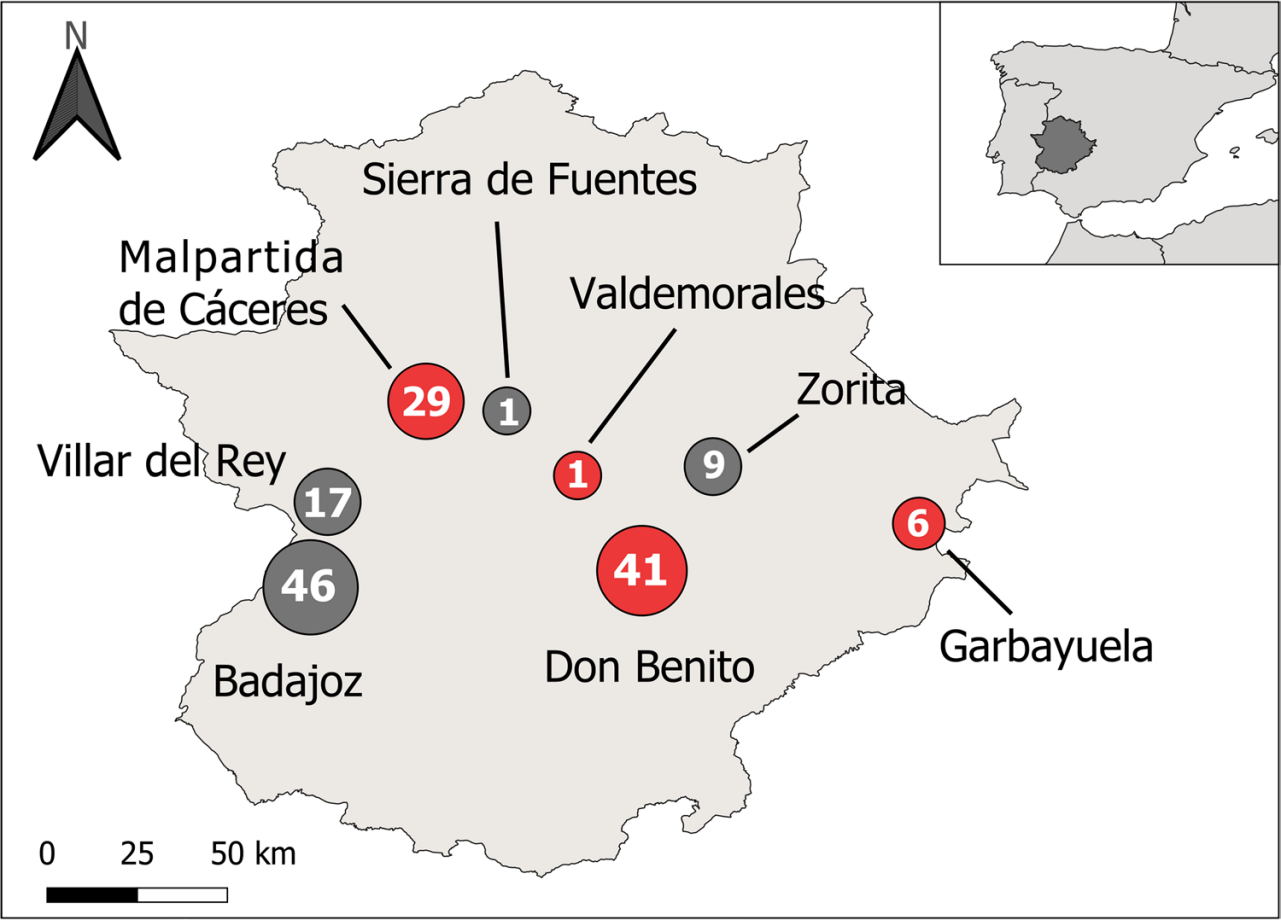
Map displaying the sampling locations in Extremadura region, Iberian Peninsula. Size of circles and the number written in them show sample size collected in each location. Locations with infected individuals found are shown in red, whereas locations without any infected individuals are shown in gray

The study was carried out across eight localities in the Extremadura region (southwestern Iberian Peninsula) (Fig. 1) in 2021. These areas were mainly composed of holm oak woodlands (*Quercus ilex* L.), grasslands, and farmlands of typically Mediterranean climate, characterized by temperate, rainy winters, and dry, hot summers (average annual temperature and rainfall 16 °C, 615 mm) (García and Mateos 2010). Samplings were conducted sporadically and non-systematically throughout the whole year (from January to December). Amphibians and reptiles were collected opportunistically by hand or with nets searching under stones used for shelter, or among aquatic vegetation. They were handled by experienced staff and held in plastic containers for on-site blood collection immediately in the field in aseptic conditions. A blood sample of each individual was taken using heparinized needles from different body areas depending on the species. Amphibian blood samples were taken from the ventral tail vein in newts and the ventral abdominal vein in frogs and toads (Allender and Fry 2008). Blood from all reptiles was collected from caudal tail vein (Sykes and Klaphake 2008), except for turtles, in which blood was drawn from the caudal sinus at the base of the tail (Polo-Cavia et al. 2010). Samples were immediately stored in 500 μl of SET buffer (0.015 M NaCl, 0.05 M Tris, 0.001 M EDTA, pH 8.0) (Sambrook et al. 1989) for subsequent molecular analysis. All the individuals were safely released and at the same point of capture after ensuring that they were in perfect condition. We obtained blood samples of 86 amphibians belonging to five species and 59 reptiles belonging to 13 species (Table 1).

**Table 1:** Samples analyzed for apicomplexan parasites (haemosporidians and haemogregarines) in amphibians and reptiles from southwestern Iberian Peninsula. “Haemos” and “Haemog” represent the number of *Haemocystidium* and haemogregarine positive sequences, respectively. Shown are also accession numbers of positive sequences in GenBank

Host species	*N*	Haemos	Haemog	Accession number
Amphibians				
Fam. Bufonidae				
* Epidalea calamita*	41			
Fam. Hylidae				
* Hyla meridionalis*	1			
Fam. Ranidae				
* Pelophylax perezi*	27			
Fam. Salamandridae				
* Pleurodeles waltl*	9			
* Triturus pygmaeus*	8			
Reptiles				
Fam. Blanidae				
* Blanus cinereus*	3			
Fam. Colubridae				
* Natrix maura*	13			
Fam. Gekkonidae				
* Hemidactylus turcicus*	1			
Fam. Geoemydidae				
* Mauremys leprosa*	3		**1**	KJ740753
Fam. Lacertidae				
* Acanthodactylus erythrurus*	2			
* Podarcis virescens*	3		**2**	ON332722, JX531954
* Psammodromus algirus*	6			
* Psammodromus occidentalis*	3			
* Timon lepidus*	5			
Fam. Lamprophiidae				
* Malpolon monspessulanus*	6		**3**	ON332723, ON332724, KC696567
Fam. Phyllodactylidae				
* Tarentola mauritanica*	10	**2**		ON458135
Fam. Scincidae				
* Chalcides bedriagai*	1			
* Chalcides striatus*	3			

### Molecular detection of blood parasite infections

Total genomic DNA from blood samples was extracted with the Thermo KingFisher purification system™, using the MagMAX™ Cell-Free DNA Isolation kit (Thermo Fisher, Ref. A32700) and PK buffer for MagMAX™-96 DNA Multi sample kit (Thermo Fisher, Ref. 4,489,111). DNA samples were screened to identify haemosporidian parasites following the protocol by Hellgren et al. (2004), as it has been previously used to identify *Haemocystidium* in reptiles (Oliveira et al. 2018; Austen et al. 2020). We used a nested polymerase chain reaction (PCR) that targets a 478-bp fragment (excluding primers) of the mitochondrial cytochrome b gene of these parasites following Hellgren et al. (2004). This procedure is based on a first PCR using primers HaemNFI (5′-CAT ATA TTA AGA GAA ITA TGG AG-3′) and HaemNR3 (5′-ATA GAA AGA TAA GAA ATA CCA TTC-3′), followed by a second PCR using primers HaemF (5′-ATG GTG CTT TCG ATA TAT GCA TG-3′) and HaemR2 (5′-GCA TTA TCT GGA TGT GAT AAT GGT-3′). To detect the presence of haemogregarine parasites, we amplified the 18S RNA gene of *Hepatozoon* spp., as it has been previously used in other studies in reptiles (Harris et al. 2011; Marzal et al. 2017). This method uses a nested PCR amplification targeting a 600 bp of the 18S rRNA gene of apicomplexan parasites initially using the primers HEMO1 (5′-TAT TGG TTT TAA GAA CTA ATT TTA TGA TTG-3′) and HEMO2 (5′-CTT CTC CTT CCT TTA AGT GAT AAG GTT CAC-3′) (Perkins and Keller 2001) and then primers HepF300 (5′-GTT TCT GAC CTA TCA GCT TTC GAC G-3′) and HepR900 (5′-CAA ATC TAA GAA TTT CAC CTC TGA C-3′) (Ujvari et al. 2004). Negative and positive controls were run with each reaction. Parasites detected by a positive amplification were purified using the Genejet PCR Purification Kit (Thermo-Fisher Scientific) and then sequenced in both directions on an ABI 3130 genetic analyzer (provided by the Service of Bioscience Applied Techniques of the University of Extremadura, SAIUEx). A consensus sequence was created by combining the two partially overlapping regions of the forward and reverse sequences of each sample with Geneious Prime 2019.2.3 (Kearse et al. 2012). Obtained consensus sequences were then compared against published ones to find the best match on GenBank using BLAST. Three different sequences matched 100% with haemogregarine parasites previously found in reptiles. The new sequences found were deposited in GenBank under the accession numbers ON458135, ON332722, ON332723, and ON332724.

The genetic relationships of the *Haemocystidum* and haemogregarine parasites were investigated separately by analyzing the haemosporidian cytochrome b and the 18S rDNA sequence divergence respectively using the program MEGA11 v. 11.0.11 (Tamura et al. 2021). All available *Haemocystidium* sequences from GenBank were included to build the phylogenetic tree. For the haemogregarines, only those found in Mediterranean hosts were selected. The *Haemocystidium* and haemogregarine phylogenetic trees were rooted with a sequence of *Plasmodium falciparum* (GeneBank M76611) and *Adelina bambarooniae* (GeneBank AF494059), respectively. We generated an alignment of 33 sequences, with a total length of 470 bp for *Haemocystidium* and an alignment of 68 sequences, with a total length of 508 bp for haemogregarines. The AIC criterion was used to choose the best model of sequence evolution and the parameters employed for our set of sequences. Maximum likelihood analyses were performed using a general time reversible model (GTR) with a Gamma distribution and invariant sites for the *Haemocystidium* sequences and a Tamura 3-parameter model (T92) with a Gamma distribution for the haemogregarine sequences. Support for nodes was estimated using the bootstrap technique (Felsenstein 1985) with 1000 replicates. Estimates of evolutionary divergences were also calculated (Supplementary material).

## Results

Of the 86 amphibians analyzed, we did not find any infected individual neither by haemosporidian nor haemogregarine parasites. On the other hand, of 59 reptiles screened, two individuals were infected by *Haemocystidium*, one individual was infected by *Haemogregarina* and five by *Hepatozoon* (Table 1).

After comparing our sequences with those published in GenBank, we found seven parasite lineages infecting sampled individuals: one *Haemocystidium*, one *Haemogregarina*, and five *Hepatozoon* lineages. From them, four parasite sequences had not been previously detected in prior studies, and therefore were considered as new haplotypes: one *Haemocystidium* sequence was detected in *Tarentola mauritanica* (ON458135), two *Hepatozoon* lineages were found infecting *M. monspessulanus* individuals (ON332723 and ON332724), and one *Hepatozoon* lineage was detected in a *P. virescens* (ON332722). The other three haplotypes (KC696567, JX531954, and KJ740753) had been found in prior studies infecting reptiles.

**Fig. 2:**
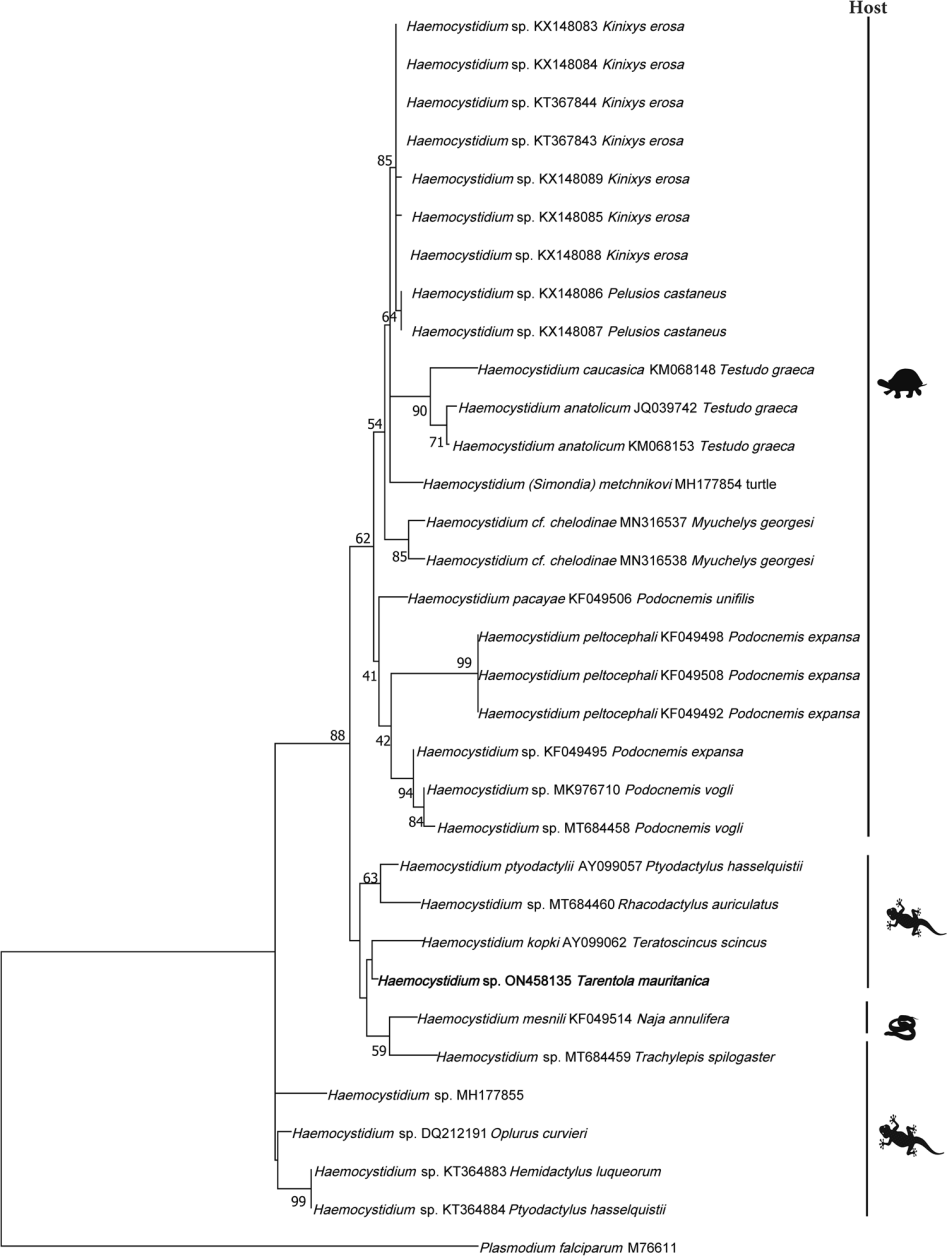
Phylogenetic relationships derived from a maximum likelihood analysis (GTR + G + I model) of *Haemocystidium* sp. mitochondrial cytochrome b sequences available in GenBank used in the phylogenetic analyses of this study. The tree was rooted with *Plasmodium falciparum* (M76611). Numbers at the branches show consensus support (%) below 40%. GenBank accession numbers and associated host are shown. New sequences found in this study are in bold. The silhouette of the host corresponds to turtles, geckos, and snakes

**Fig. 3:**
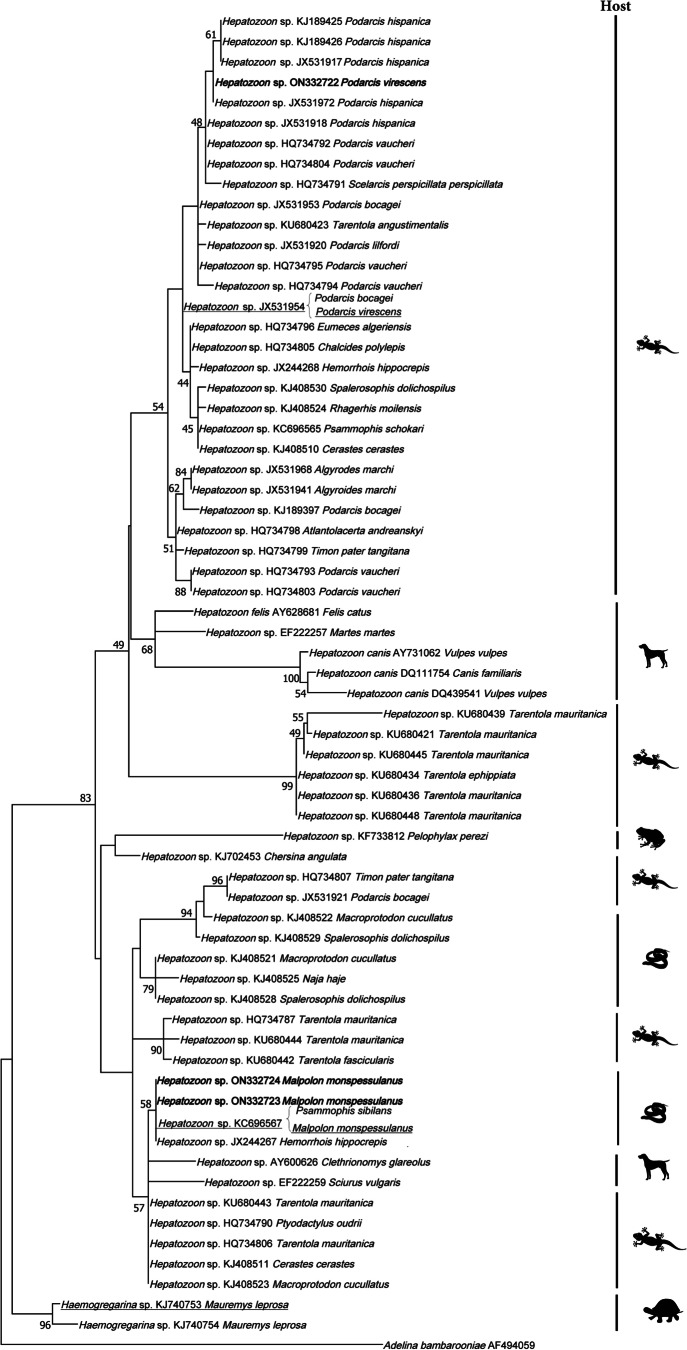
Phylogenetic relationships derived from a maximum likelihood analysis (T92 + G model) of a representative set of haemogregarine 18S rDNA sequences available in GenBank used in the phylogenetic analyses of this study. The tree was rooted with *Adelina bambarooniae* (AF494059). Numbers at the branches show consensus support (%) below 40%. GenBank accession numbers and associated host are shown. New sequences found in this study are in bold and those previously reported in other host species are underlined. The silhouette of the host corresponds to anurans, (geckos and lizards), mammals, snakes, and turtles

The *Haemocystidium* phylogenetic analyses revealed the affiliation of the new haplotype from *T. mauritanica* with *Haemocystidium* sequences from geckos and snakes, which were separated from lineages found in chelonians (Fig. 2). The haemogregarine phylogenetic analyses revealed that the new *Hepatozoon* lineages from *M. monspessulanus* were closely related to other *Hepatozoon* sequences found in Iberian and North African snakes forming an individual clade. The new lineage obtained from *P. virescens* formed a clustered with other *Hepatozoon* lineages found in *Podarcis* spp. (Fig. 3).

## Discussion

The knowledge of apicomplexan parasite diversity in amphibians and reptiles has increased notably in the last decade due to the use of molecular methods (O’Dwyer et al. 2013; Tomé et al. 2014; Harris et al. 2015). Here, we analyzed 145 individuals of 18 amphibian and reptile species from the Iberian Peninsula to characterize the genetic diversity of haemosporidian and haemogregarine parasites. None of the 86 individuals from five amphibian species were infected by these blood parasites. In contrast, of the 59 reptiles analyzed, one *Haemogregarina*, five *Hepatozoon*, and one *Haemocystidium* lineages were found infecting four reptile species. Notably, more than a half of these haplotypes had not been previously recorded in former studies. Moreover, our outcomes also revealed six new reptile—parasite records. These findings highlight the great unexplored diversity of apicomplexan parasites in reptiles from this region.

Amphibians are hosts of a wide variety of hemoparasites including *Haemogregarina*, *Hepatozoon*, *Lankesterella*, or *Schellackia* among others (Muriel et al. 2021). The presence of *Haemoproteus* in these animals is very unusual, having been identified only three species to date (Fantham et al. 1942), while haemogregarine parasites, and *Hepatozoon* species particularly, are more frequently recorded (e.g., Leal et al. 2009; Netherlands et al. 2015; Maia et al. 2016b). However, although some haemogregarine infections have been found in reptiles from nearby localities (e.g., Marzal et al. 2017; this study), none of the amphibians analyzed in this study was infected. The absence of infected amphibians could be explained by the methodological approach used. Seabra-Babo et al. (2015) failed to amplify DNA of any amphibian haemogregarines using the Hep primers (Ujvari et al. 2004), while they were clearly identified on visual screening of blood smears. On the other hand, Harris et al. (2013) and Maia et al. (2016b) detected some positive amphibians infected by *Hepatozoon* using the same primers. Therefore, based on these contradictory results, Haem and Hep primers may not be effective in detecting haemogregarine and haemosporidian parasites in amphibians. Alternatively, these differences could be also explained by other factors. While reptiles are mostly terrestrial, amphibians spend much of their time into the water. The absence of infected individuals in our samples could be due to the absence of appropriate aquatic vectors infecting amphibians in the study area or due to the short exposure time to terrestrial vectors (Ball 1967; Barta and Desser 1984; Bennett et al. 1992). In addition, the available vectors or parasite haplotypes in the area may have a high host-specificity infecting only reptile species instead of amphibians (Apperson et al. 2004; Maia et al. 2016b; Fecchio et al. 2019). Finally, amphibians can recognize both physical and chemical cues from some potential parasites, avoiding sites where vector or parasite densities are high (Kiesecker and Skelly 2000; Ferguson and Smith 2012). Nevertheless, our results should be taken with caution due to low sample sizes collected *per* species and which make it difficult to draw accurate conclusions. This highlights the need for studies with a larger sample size or with other specific primers in order to corroborate the absence or presence of parasites in Iberian amphibians.

The genus *Plasmodium* was not found in this study, in contrast with other studies in reptiles (e.g., Matta et al. 2018; Harris et al. 2019). On the contrary, two lineages of *Haemocystidium* sp. and six of *Hepatozoon* sp. were found among reptiles studied. The reports of *Haemocystidium* sp. worldwide are still limited in literature, being found almost exclusively infecting some gecko and turtle species (Telford 2005; Javanbakht et al. 2015; Maia et al. 2016a). Thus, to our knowledge, it represented the first report of *Haemocystidium* in western Europe and the first in the species *Tarentola mauritanica*. The phylogenetic analysis was similar to those previously described by González et al. (2019) and Austen et al. (2020), with a clade formed by parasites found in chelonians and other clade formed by parasites found in lacertids and snakes. The new lineage ON458135 found from *T. mauritanica* could be placed together with this second clade with haplotypes from other gecko and a snake species.

Six haplotypes of haemogregarines from six infected reptiles were found in this study. Haemogregarines are frequently reported infecting reptiles worldwide, with usually high prevalence (e.g., Ujvari et al. 2004; Maia et al. 2012, 2016b). In spite of the low number of individuals collected per species, our results seem to show that the occurrence of haemogregarines varied among species, as we only detected parasites in three of the thirteen species studied. Differences in susceptibility to infection among species could be associated with host immune defenses that can lead to prevent or better tolerate the infection (Klein 2004; Lindström et al. 2004). Alternatively, both the parasites and the vectors that transmit them may have a high degree of specificity for certain species of reptiles (Maia et al. 2016b; Fecchio et al. 2019). This may explain why some host species were more likely to be infected than others. Moreover, the host-related variables, for example, age or size (Brown et al. 2006; Salkeld and Schwarzkopf 2005), methodological aspects (Harris et al. 2011), or ecological factors (Sehgal et al. 2011; Gupta et al. 2013), must be also considered when analyzing infection levels from different species and from different geographical areas. However, as in amphibians, the limitations of these results have to be taken into account since some host species were represented by only one individual and because of the low sample size collected *per* species the probabilities of detecting infection were generally low and probably a bit random.

Among the infected species, we found two *Hepatozoon* haplotypes infecting the lacertid *Podarcis virescens* in this study. One of them (JX531954) was previously reported infecting *P. bocagei* from Tanes (Spain) (Maia et al. 2012). Phylogenetic analyses revealed that the haplotype was closely related to other North African lacertid haplotypes. The other new isolate (ON332722) fell in a group with other *Podarcis* species from North Africa and the Iberian Peninsula (*P. hispanica* and *P. vaucheri*). Our findings represented the first report of *Hepatozoon* parasites infecting the species *P. virescens*.

Moreover, the haplotypes found infecting *Malpolon monspessulanus* in this study also represented the first report of *Hepatozoon* sp. in this species. According to the phylogenetic analyses, the two new isolates appeared to be closely related to each other forming part of a group with other haplotypes found in snakes from North Africa and the Iberian Peninsula. The third haplotype identified was previously reported by Tomé et al. (2013) from *Psammophis sibilans* in North Africa and was also included in the snake group previously mentioned. This result followed the idea that some *Hepatozoon* parasites are not host-specific (Maia et al. 2016b; Tomé et al. 2021). The apparent switch of host genus may be facilitated by the hosts similarity (both are snakes), and therefore it is assumed that they will have more similar immune defenses to deal with (Medeiros et al. 2013; Maia et al. 2016b; Clark and Clegg 2017). Furthermore, this may be more likely to occur when these hosts share a habitat (Maia et al. 2016b). However, both records take place in different regions separated by a geographical barrier. In this sense, other studies with avian haemosporidians have reported the same patterns across the Strait of Gibraltar, finding the *Plasmodium* lineage LK6 (H158) in birds of Spain and Africa (Mata et al. 2015).

Finally, the *Haemogregarina* haplotype from *Mauremys leprosa* found here belonged to a previously reported lineage from the same species in a nearby locality (Marzal et al. 2017) and was very closely related to another *M. leprosa* haplotype of the same locality.

In conclusion, despite the low number of individuals infected by haemogregarine parasites, a high number of new lineages were detected, pointing out the lack of knowledge about the diversity of these parasites in the Iberian Peninsula. It is for this reason that much information is still required. More studies with higher sample sizes are needed to understand the true diversity of parasites in this area and the differences in prevalence between hosts. The knowledge of this information can be critical to understand their impact on biodiversity and can also have implications for conservation, especially in host groups as threatened and vulnerable as amphibians and reptiles.

## Supplementary Information

Below is the link to the electronic supplementary material.

Supplementary file1 (XLS 95 KB)
